# Account for the Full Extent of Esophagus Motion in Radiation Therapy Planning: A Preliminary Study of the IRV of the Esophagus

**DOI:** 10.3389/fonc.2021.734552

**Published:** 2021-11-25

**Authors:** Aihui Feng, Hengle Gu, Hua Chen, Yan Shao, Hao Wang, Yanhua Duan, Ying Huang, Tao Zhou, Zhiyong Xu

**Affiliations:** ^1^ Department of Radiation Oncology, Shanghai Chest Hospital, Shanghai Jiao Tong University, Shanghai, China; ^2^ Shandong Cancer Hospital and Institute, Shandong First Medical University and Shandong Academy of Medical Sciences, Jinan, China

**Keywords:** 4DCT, lung cancer, esophagus motion, dosimetry, internal organ at risk volume

## Abstract

**Objective:**

Accounting for esophagus motion in radiotherapy planning is an important basis for accurate assessment of toxicity. In this study, we calculated how much the delineations of the esophagus should be expanded based on three-dimensional (3D) computed tomography (CT), four-dimensional (4D) average projection (AVG), and maximum intensity projection (MIP) scans to account for the full extent of esophagus motion during 4D imaging acquisition.

**Methods and Materials:**

The 3D and 4D CT scans of 20 lung cancer patients treated with conventional radiotherapy and 20 patients treated with stereotactic ablative radiation therapy (SBRT) were used. Radiation oncologists contoured the esophagus on the 3DCT, AVG, MIP and 25% exhale scans, and the combination of the esophagus in every phase of 4DCT. The union of all 4D phase delineations (U4D) represented the full extent of esophagus motion during imaging acquisition. Surface distances from U4D to 3D, AVG, and MIP volumes were calculated. Distances in the most extreme surface points (1.5 cm most superoinferior, 10% most right/left/anteroposterior) were used to derive margins accounting only for systematic (delineation) errors.

**Results:**

Esophagus delineations on the MIP were the closest to the full extent of motion, requiring only 6.9 mm margins. Delineations on the AVG and 3D scans required margins up to 7.97 and 7.90 mm, respectively. The largest margins were for the inferior, right, and anterior aspects for the delineations on the 3D, AVG, and MIP scans, respectively.

**Conclusion:**

Delineations on 3D, AVG, or MIP scans required extensions for representing the esophagus’s full extent of motion, with the MIP requiring the smallest margins. Research including daily imaging to determine the random components for the margins and dosimetric measurements to determine the relevance of creating a planning organ at risk volume (PRV) of the esophagus is required.

## 1 Introduction

During the radiotherapy of intrathoracic tumors (such as lung cancer, lymphoma, thymoma, esophageal cancer, etc.), the esophagus inevitably receives a certain degree of radiation dose. Though the existing treatment planning system can constrain the volume and dose of irradiated normal tissues, the esophagus cannot be completely excluded from the irradiation field. Acute radiation esophagitis (AE) is a common complication (1). AE leads to the decline in the quality of life and the interruption of treating course, thereby affecting the curative effect and may even cause death (2, 3). In recent years, concurrent chemoradiotherapy or hyperfractionated radiotherapy has improved the long-term survival rate and local control rate. These gains, however, come at a cost of increased toxicity, especially esophagitis (4–6).

In the process of radiotherapy planning, the prediction of AE is assessed by the dosimetric parameters such as V_40_ and V_60_ of the esophagus (7, 8), but the premise of this prediction model is the accurate delineation of the esophagus. As an important organ-at-risk (OAR) for intrathoracic radiotherapy, esophagus is usually outlined on the 3-dimensional computer tomography (3DCT) or 4-dimensional (4D) average projection (AVG). However, the above two delineation methods are unable to capture the full range of esophagus motion, including esophageal motion, peristalsis, breathing motion, and heart motion, which affects the accuracy of the dose calculation. Therefore, the accurate delineation of the esophagus has a great effect on the protection of the esophagus in radiotherapy.

Scholars conducted research on the motion of the esophagus during radiotherapy. Nardone et al. (9) confirmed that the internal organ at risk volume (IRV) of the esophagus contoured in all respiratory phases was 25% larger than the esophagus conventionally outlined. Gao et al. (10) found that in a phase I dose escalation study of accelerated radiotherapy with concurrent chemotherapy for locally advanced lung cancer, the intrafractional center shifts were 0.6 ± 0.4, 0.7 ± 0.7, and 0.9 ± 0.7 mm for the upper, middle, and lower esophageal regions, respectively. Based on their findings, we believe that the accurate delineation of the esophagus needs to consider the peristalsis and movement of the esophagus, as well as breathing and heart movements.

For intrathoracic radiotherapy, the treatment plan is performed on CT. Due to the time difference between respiratory motion and CT scan, 3DCT images may be randomly located in a certain respiratory phase or interlaced between adjacent respiratory phases, therefore it cannot reflect the motion information of the target and OARs in different phases. Cine mode of 4DCT can collect images of the entire respiratory cycle, and generates multiple time-phased images according to the respiratory signal, which can reflect the whole breathing movement of the OARs and the real movement trajectory of the target.

In this study, we attempted a 4D method of esophagus delineation and evaluated how much the delineations based on the 3D, AVG, and the maximum intensity projection (MIP) scan should be extended (using margins) to obtain a comprehensive consideration of esophageal movement, respiratory movement, peristalsis, and heart movement. The margins we derived can be used to create an IRV for the esophagus and provide data for PRV.

## 2 Material and Methods

### 2.1 Patient Data

We used 3D and 4DCT scans of 20 lung cancer patients who received intensity-modulated radiation therapy (IMRT) and 20 lung cancer patients treated with stereotactic ablative body radiation (SBRT) at the Shanghai Chest Hospital, between January 2019 and March 2021. Institutional approval was granted to use the data (the committee’s reference number: KS1974). The 4DCT scans consisted of nine phases, corresponding to 0, 25, 50, and 75% expiration and 100, 75, 50, 25, and 0% inspiration. According to the reconstruction of the 4DCT scans, AVG and MIP scans were derived. The AVG image results from averaging all phase CT scans, and the MIP is derived by finding the maximum intensity at all voxel locations over all phases. MIP projections reflect the highest data value encountered along the viewing ray for each pixel of volumetric data, giving rise to a full intensity display of the brightest object along each ray on the projection image. The AVG and MIP scans were generated in MIM software (MIM Maestro, version 7.0.4, MIM Software Inc, Cleveland, OH).

### 2.2 Manual Delineations

An experienced radiation oncologist delineated the esophagus in 3D, AVG, MIP scans and an arbitrarily chosen phase, 25% exhale. The delineation was contoured in MIM, according to the principles of RTOG 1106. In addition, the esophagus delineations were revised by another observer.

### 2.3 Union of 4D Phase Delineations Represents the Esophagus’s Full Extent of Motion

The phases of 4DCT scans provide snapshots of the esophageal movement with the respiratory cycle, so if we have a sketch of the esophagus in each 4D phase, the combination of these time-phased esophageal profiles will include the full extent of motion during image acquisition. Since it is time-consuming to contour the esophagus on every phase, we use MIM software to make full-time mapping of the esophagus by continuous deformation registration. The manual outline of the 25% in phase was propagated to all the remaining phases. Finally, the esophagus’s full extent of motion was then defined as the union of all these propagated delineations. We refer to the union of all 4D phases as U4D_Auto_.

### 2.4 Contour Propagation Evaluation

The continuous deformation registration method of MIM software for delineation has not been clinically verified. To determine whether the propagation profile is an effective way to delineate the full extent of motion, a pre-experiment was carried out. We manually contoured the esophagus in all remaining 4D phases for 10 patients and referred to as U4D_Manual_. For the next step, U4D_Auto_ and U4D_Manual_ were compared by the volume, Hausdorff Distance (HD), Mean Distance to Agreement (MDA), Dice coefficient, and Jaccard coefficient. These metrics were calculated using the formula below:


(1) HD (A,B)=max(h(A,B),h(B,A))

Where h (A, B) and h (B, A) are respectively defined in Eqs. (2) and (3)


(2) $$h(A,B)=\underset{a\in A}{\max}\left\{\underset{b\in B}{\min}\left||a-b|\right|\right\}$$

(3) $$h(B,A)=\underset{b\in B}{\max}\left\{\underset{a\in A}{\min}\left||b-a|\right|\right\}$$

(4) $$Dice\;coefficient=\frac{2|V_{A}\cap V_{B}|}{\left|V_{A}|+|V_{B}\right|}$$

(5) $$Jaccard\;coefficient=\frac{\left|A\cap B\right|}{\left|A\cup B\right|}$$

where A represents the U4D_Auto_, and B represents the ground truth U4D_Manual_ created by an experienced radiation oncologist. MDA is the mean distance between the surfaces of both volumes, with a value of 0 representing perfect agreement. HD, MDA, Dice coefficient, and Jaccard coefficient were all generated in MIM software.

If U4D_Auto_ failed any of the above assessments, then for accuracy, all U4D_ESO_ were manually outlined by an experienced radiation oncologist in this study.

### 2.5 The Calculation of Margins

In order to determine the extent to which the esophageal contour needs to be extended in 3D, AVG, and MIP scans, the bidirectional local distance method (11) was used to calculate the distance from the above esophageal surface to the U4D_ESO_ surface and based on McKenzie’s method (12) to calculate the margin of the esophagus.

#### 2.5.1 Distances to U4D

To calculate the margin, we collected and summarized the distances on the right, left, anterior, posterior, inferior, and superior aspects of the U4D_ESO_ surface.

The coordinates of all voxels of esophageal delineation were collected from the RTstructure DICOM file by MATLAB (version 2018a). The distances on the right, left, anterior, posterior, inferior, and superior aspects of the esophagus surface were the differences of voxel coordinates. These aspects were represented by the most extreme points of ESO_3D_, ESO_AVG_, ESO_MIP_, and U4D_ESO_.

**Figure 1:**
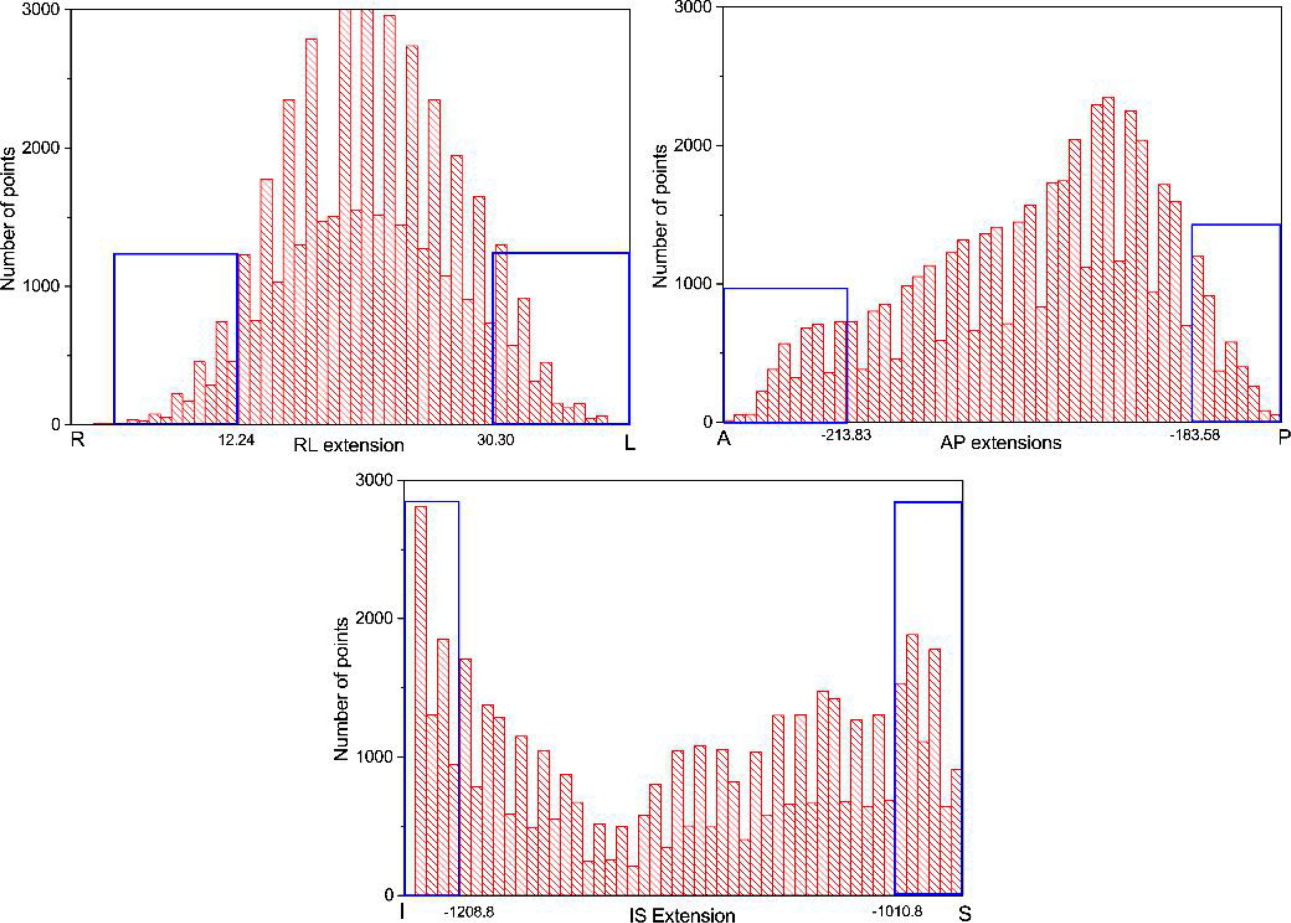
The histograms used to select the most inferior/superior, anterior/posterior, and right/left points. The voxel points in the blue frame are the extreme points.

The most inferior points were defined as the points lying in the five lowest slices. The threshold used was the lowest IS coordinate +15 mm. Conversely, the most superior points were defined as the points lying in the five highest slices. The threshold was set to the highest IS coordinate −5 mm. From the points not selected in the previous steps, the most anterior and posterior points were defined as the points lying in the 10% most anterior and most posterior region; The extremum points in RL direction are the 10% most right and most left voxels after excluding the extremum points in AP and IS directions. **Figure 1** shows the selection of extreme points. The in-house code for collecting extreme points is presented in **Appendix A**.

#### 2.5.2 Margin Components, M and Σ

The overall mean (or group systematic) error, M, as well as the systematic error, Σ, were determined. The overall mean error, M, was defined as the mean of these means over all patients. Conversely, the systematic error, Σ, was defined as the standard deviation of these means over all patients.

#### 2.5.3 The Calculation of Margin

We used the method proposed by McKenzie to calculate the margin of the esophagus. McKenzie’s formula is shown in **Appendix B**. The overall average error M and the system error Σ were selected from the main axis direction.

### 2.6 Dosimetric Analysis

For the dosimetric analysis, we investigated two treatment methods, IMRT and SBRT. For the above four kinds of delineation, ESO_3D_, ESO_AVG_, ESO_MIP_, ESO_U4D_, D_max_, D_mean_, V_20_, V_30_, V_35_, V_40_, V_45_, V_50_, V_55_, V_60_, and NTCP of IMRT group were calculated respectively. For SBRT patients, in addition to the above dosimetric parameters, V_19.5 Gy_ is added, which is a dose parameter selected based on Timmerman’s recommended dose limit (13) of hypofractionated radiation therapy.

### 2.7 Statistical Analysis

Nonparametric statistical tests were used. Student t-test was used as a paired test of the significance of differences in signals between the dose metrics of ESO_3D_, ESO_AVG_, ESO_MIP_, and U4D_ESO_. P-values less than 0.05 were considered statistically significant.

## 3 Result

### 3.1 Pre-Experiment: The Accuracy Assessment of U4D_Auto_

Through volume, HD, MDA, Dice, and Jaccard coefficients, this study compared all the 4D phase manual delineation of the esophagus for 10 patients with the automatic delineation of continuous deformation registration. It can be seen in **Table 1** that U4D_Auto_ and U4D_Manual_ are quite different. Except for one patient, the volume of U4D_Auto_ is smaller than U4D_Manual_. In addition, although the Dice coefficient of U4D_Auto_ and U4D_Manual_ is high, the HD distance is quite large (>8 mm), and HD distance of a patient is up to 20.14 mm. Therefore, we conclude that the existing continuous deformation registration algorithm of MIM is not a robust alternative to outline U4D_ESO_. Even though the continuous deformation registration of MIM can achieve a Dice coefficient of 0.91 in the delineation of individual patients with manual delineation, the HD is unacceptable, and the volume of the U4D_Auto_ is significantly smaller than U4D_Manual_ (p <0.001).

**Table 1:** Volume, HD, MDA, Dice and Jaccard coefficient of U4D_Auto_ and U4D_Manual_ for 10 patients.

# Patient	Volume (cc)		HD (mm)	MDA (mm)	Dice	Jaccard
	U4D_Auto_	U4D_Manual_				
**1**	41.90	47.09	12.14	0.81	0.87	0.76
**2**	48.18	58.29	10.81	0.77	0.88	0.78
**3**	58.13	61.58	9.39	0.66	0.91	0.82
**4**	45.83	53.57	15.15	0.82	0.85	0.74
**5**	53.37	56.77	9.63	0.93	0.84	0.72
**6**	38.13	48.84	8.30	0.86	0.84	0.73
**7**	47.48	54.15	13.19	0.76	0.87	0.76
**8**	61.58	76.97	20.14	0.98	0.86	0.75
**9**	71.88	79.85	12.25	0.90	0.87	0.76
**10**	60.86	60.21	8.43	0.60	0.90	0.82
**Mean ± SD**	52.73 ± 10.37	59.73 ± 10.87	11.94 ± 3.62	0.81 ± 0.12	0.87 ± 0.02	0.77 ± 0.03

In this study, for accuracy, the U4D of the esophagus for all forty patients were contoured manually by an experienced radiation oncologist. To facilitate understanding, the U4D of esophagus will be referred to as ESO_U4D_.

### 3.2 Contour Variation Between ESO_3D_, ESO_AIP_, ESO_MIP,_ and ESO_U4D_

**Figure 2:**
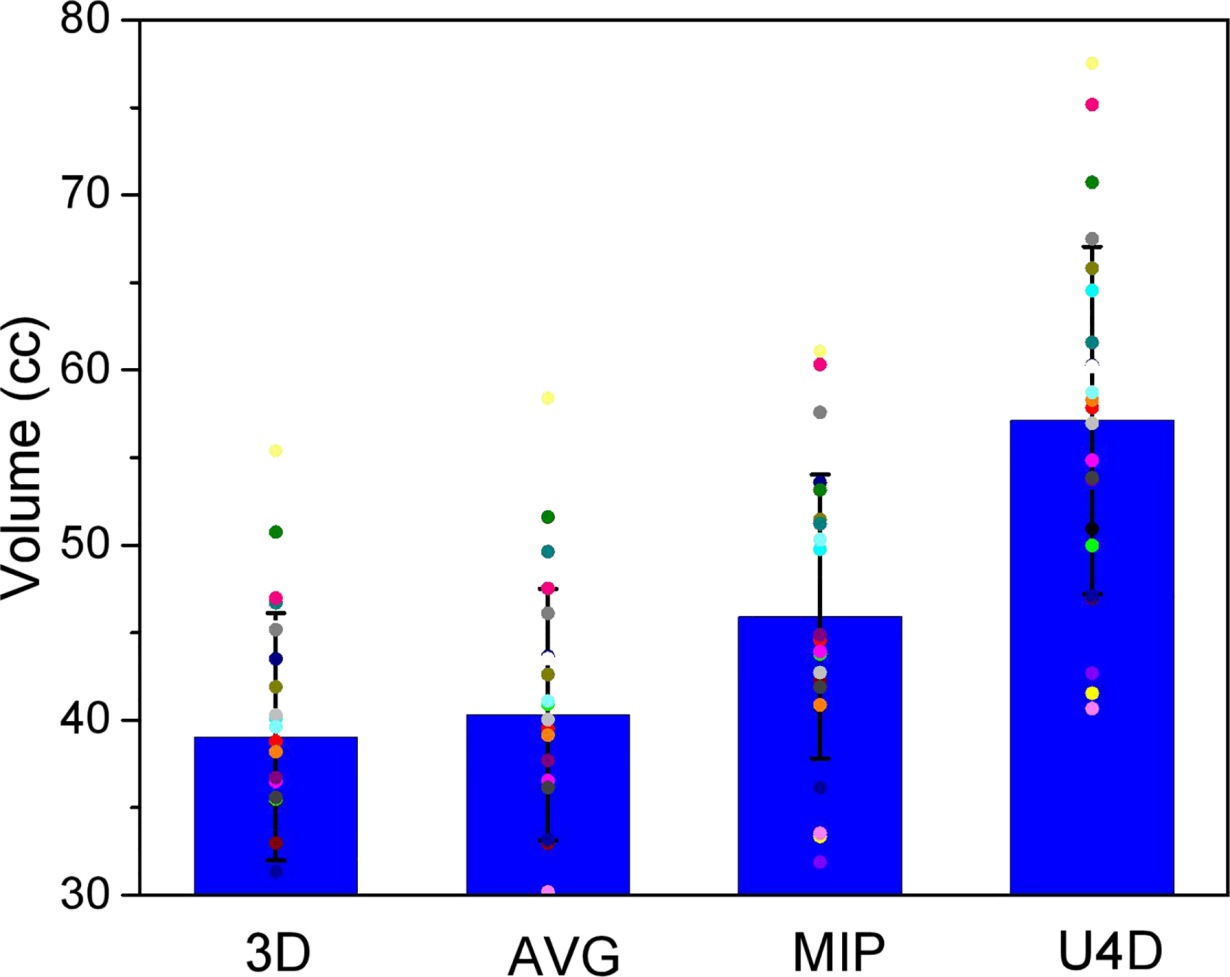
The volume of the heart delineations for the 3-dimensional, average projection, and maximum intensity projection scans and the full extent of motion (U4D).

**Figure 2** shows the median value of esophagus volume on the 3D, AVG, and MIP scan for 40 patients, as well as the volumes of the corresponding U4D. Our results confirm that in 4DCT, ESO_U4D_ are much larger than other esophagus delineations (46.70% for ESO_3D_, 42.01% for ESO_AIP_, and 24.69% for ESO_MIP_).

The HD, MDA, Dice, and Jaccard coefficients between three delineations and U4D of 40 patients are reported in **Table 2**. The trends of the four coefficients are consistent, showing the degree of similarity between the three outlines and U4D are: 3D < AVG < MIP.

**Table 2:** Mean HD, MDA, Dice, and Jaccard coefficients between 3D, AVG, MIP, and U4D contours.

Index	3D *vs* U4D (mean **±** SD)	AVG *vs* U4D (mean **±** SD)	MIP *vs* U4D (mean **±** SD)
**HD (mm)**	16.65 ± 6.38	15.32 ± 7.09	13.62 ± 6.04
**MDA (mm)**	1.43 ± 0.47	1.28 ± 0.45	0.84 ± 0.38
**Dice**	0.76 ± 0.05	0.79 ± 0.05	0.86 ± 0.05
**Jaccard**	0.62 ± 0.06	0.65 ± 0.07	0.76 ± 0.08

### 3.3 Distances to U4D

**Figure 3:**
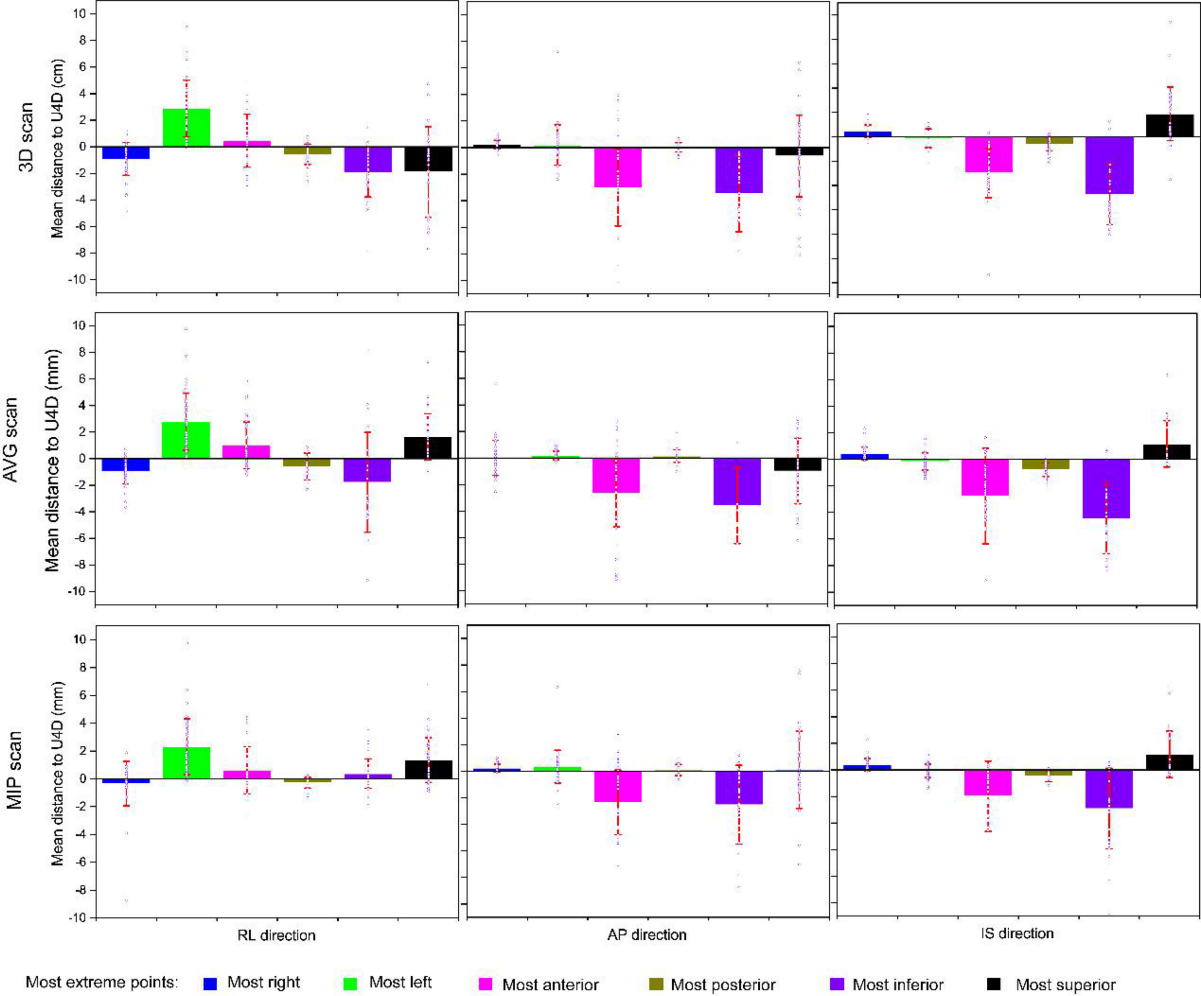
Mean distances to the full extent of motion (U4D) for the delineations on the 3-dimensional, average projection, and maximum intensity projection scans (rows). The columns report the distance components: the right-left (RL), anteroposterior (AP), and anterosuperior (IS). The square marker represents the mean of means (M); the error bar represents standard deviation (i.e., Σ). The blue hollow dots represent the individual mean distances per patient.

The mean distances (and standard deviations) to the full extent of motion, U4D, for the extreme points, for the delineations on the 3D, AVG, and MIP scans are presented in **Figure 3**. **Table 3** shows M, and Σ, and the data of the main axis are displayed in bold. The largest distances were found for the delineations on the 3D scan. Distances for the MIP delineations are lower than those for the 3D and AVG delineations.

**Table 3:** Overall mean errors and systematic errors for all extreme points and distance components for delineations on the 3D, AVG and MIP scans.

	Overall mean error (M, mm)					
	R	L	A	P	I	S
**Delineations on 3D scans**						
**RL distance**	**−0.92**	**2.88**	0.45	**−**0.57	**−**1.94	**−**1.90
**AP distance**	0.22	0.19	**−3.01**	**0.20**	**−**3.45	**−**0.64
**IS distance**	0.46	**−**0.13	**−**2.98	**−**0.63	**−4.73**	**1.89**
**Delineations on AVG scans**						
**RL distance**	**−0.96**	**2.79**	1.00	**−**0.59	**−**1.78	1.64
**AP distance**	0.20	0.01	**−2.65**	**0.15**	**−**3.57	**−**0.98
**IS distance**	0.43	**−**0.16	**−**2.8	**−**0.79	**−4.48**	**1.67**
**Delineations on MIP scans**						
**RL distance**	**−0.34**	**2.32**	0.61	**−**0.28	0.37	1.34
**AP distance**	0.23	0.34	**−2.35**	**0.10**	**−**2.52	0.10
**IS distance**	0.39	**−**0.08	**−**1.98	**−**0.47	**−2.91**	**1.16**
	**Systematic error (Σ, mm)**					
	**R**	**L**	**A**	**P**	**I**	**S**
**Delineations on 3D scans**						
**RL distance**	**1.24**	**2.13**	1.99	0.76	1.84	3.41
**AP distance**	0.33	1.53	**2.90**	**0.35**	2.89	3.08
**IS distance**	0.52	0.76	2.02	0.55	**2.49**	**2.19**
**Delineations on AVG scans**						
**RL distance**	**0.94**	**2.15**	1.76	1.01	3.76	1.73
**AP distance**	0.32	1.32	**2.54**	**0.47**	2.87	2.46
**IS distance**	0.50	0.65	3.59	0.51	**2.63**	**1.81**
**Delineations on MIP scans**						
**RL distance**	**1.57**	**2.00**	1.71	0.38	1.06	1.61
**AP distance**	0.30	1.23	**2.43**	**0.42**	2.97	2.91
**IS distance**	0.45	0.50	2.64	0.41	**3.04**	**1.75**

The largest component per extreme point group is emphasized in bold font. Notice that the sign indicates direction: For RL distance <0 indicates towards the right and >0 towards the left. For AP distance, *<*0 indicates towards anterior and >0 towards posterior. Conversely, for IS distance, *<*0 indicates towards inferior and >0 towards superior.

The behavior of the largest distance components is as expected: toward the right for the right-most points, toward the left for the left-most points, toward the anterior for the anterior-most points, toward the posterior for the posterior-most points, toward the inferior for the inferior-most points, and toward the superior for the superior-most points. We derived margins using only these main axes.

### 3.4 Margins

M and Σ in the main axis direction were used to calculate the margin of the esophagus, taking only delineation error into account. **Table 4** shows the margins obtained using the technique proposed by McKenzie et al. It can be seen that the margin of the 3D scan is the largest (≥0.6 mm for all components), followed by the AVG and MIP. From this perspective, the posterior is the smallest of all contours (≤1 mm), which may be affected by the esophagus-bone interface. On the contrary, the inferior margin is the largest of all contours (≥6 mm), followed by anterior direction (≥5 mm).

**Table 4:** Overall mean error M and systematic error Σ used to derive margins for delineations on the AVG and MIP scan using the full extent of motions as ground truth.

Delineations on	Margin components (mm)					
	R	L	A	P	I	S
**Margin 1D**						
**3D scans**	2.53	5.64	6.78	0.66	7.97	4.74
**AVG scans**	2.18	5.59	5.95	0.76	7.90	4.02
**MIP scans**	2.38	4.92	5.51	0.65	6.86	3.44
**Margin 2D**						
**3D scans**	3.65	7.57	9.39	0.97	10.21	6.71
**AVG scans**	3.03	7.52	8.24	1.18	10.27	5.65
**MIP scans**	3.79	6.72	7.70	1.02	9.60	5.01
**Margin 3D**						
**3D scans**	4.02	8.21	10.26	1.08	10.96	7.37
**AVG scans**	3.31	8.17	9.0	1.33	11.06	6.20
**MIP scans**	3.79	6.72	8.43	1.02	9.60	5.01

### 3.5 Dosimetric Impact

The IMRT and SBRT groups’ dosimetric parameters were compared between ESO_3D_, ESO_AVG_, ESO_MIP_, and ESO_U4D_. The prescription for the IMRT was 60 Gy/30fx, and was 50 Gy/5fx for the SBRT group.

**Table 5** lists the statistically significant dose parameters of the esophagus, others are not listed. In the IMRT and SBRT groups, the D_max_, D_mean_, and NTCP of ESO_3D_, ESO_AVG_, ESO_MIP_, and ESO_U4D_ were significantly different (p <0.01). In the IMRT group, V_20Gy_ is also statistically different. In addition, in this study, the number of patients whose D_max_ of ESO_U4D_ exceeded the dose limit was counted. It can be seen that there are four people in the IMRT group who exceed the limit, but there is no over-limit in the SBRT group.

**Table 5:** List of dose parameters that are statistically different from U4D_ESO_.

	sIMRT				SBRT			
	3D	AVG	MIP	U4D	3D	AVG	MIP	U4D
**D_max_(Gy)**	52.94	54.04	54.80	56.23	9.58	9.58	9.89	10.07
**D_mean_(Gy)**	12.35	12.64	12.95	12.41	1.41	1.38	1.38	1.31
**NTCP (%)**	0.51	0.54	1.07	1.26	0.13	0.13	0.13	0.14
Nb of patients exceed D_max_*	0	0	2	4	0	0	0	0
**V_20Gy_ (%)**	21.13	21.88	22.57	21.59	/	/	/	/

*In the IMRT group, the D_max_ limit was 64.2 Gy; in the SBRT group, the D_max_ limit was V_19.5 Gy_ > 5 cc.

## 4 Discussion

In this study, all the movements of the esophagus, including esophageal movement, peristalsis, respiratory movement, and cardiac motion, were taken into account to calculate the systematic error expansion (using margins) of the contour based on 3DCT, AVG, and MIP scans. It was found that the continuous deformation registration of MIM for the esophageal delineation in the full-time phase has a large HD distance and significant difference in volume when compared to manual delineation, which was not a robust method for automatic delineation of the esophagus in the 4D full-time phase. In this paper, we compared the esophagus delineations of 3DCT, AVG, MIP, and the U4D_ESO_ which includes the full range of motion of the esophagus. In terms of volume, HD, MDA, Dice, and Jaccard coefficients, the differences between the delineations and U4D_ESO_ were: 3D > AVG > MIP. The esophagus’s IRV expansion of 3DCT, AVG, and MIP is calculated by the bidirectional local distance method. The three aspects with larger distances on the main axis were right, anterior, and inferior, respectively. The data adopted in this research were lung cancer patients, but the IRV expansion is also applicable to other intrathoracic tumors in which the esophagus is endangering.

To the best of our knowledge, using U4D to calculate systematic errors accounts for the breathing cycle and cardiac contraction, esophagus movement, and peristalsis is a novel strategy. In this study, only the contour error was considered to calculate the esophagus margin. It was found that the posterior expansion was the smallest (≤0.7 mm), which may be due to the interface with the bone. The largest expansion is on the inferior aspect which is mainly caused by the peristalsis of the esophagus. Besides, the margin of the anterior aspect is quite large (≥5 mm), which is induced by the traction of the lung motion in the anterior direction.

**Table 5** shows the dosimetric parameters of esophagus delineations in 3D, AVG, and MIP scans, which are statistically different from those of U4D. For point dose parameters such as D_max_, we can see the important application of U4D in radiotherapy planning. Approximately 20% of patients have a higher point dose on U4D that cannot be fully detected by 3D and AVG scans. The statistical difference of NTCP also showed that the commonly used 3DCT contouring underestimated the radiation injury probability of the esophagus. For patients with conventional radiotherapy, U4D delineation considering the full extent of motion can demonstrate the D_max_ of the esophagus more accurately. For patients with hyperfractionated radiotherapy, there is no difference in the dose limit data in this study, which is mainly due to the large distance between the target and the esophagus. In clinical radiotherapy, if the esophagus is close to the target, the motion of the esophagus in SBRT still needs to be cautious. The margin of esophageal IRV in this paper can provide data for the protection of the esophagus in radiotherapy planning.

The motion of the esophagus is relatively complex, including esophagus movement, respiratory movement, esophagus peristalsis, traction of the heart and big blood vessel pulsation, etc. With the development of precision radiotherapy, not only the target needs to be delineated on the 4D full-time phase to account for the respiratory movement, but also the OARs. MIP retains the maximum CT value of the same spatial position in all phases. When being enhanced, the tissues seen in MIP are composed of motion range. As a result, MIP can better reflect the individualized motion range of tumors and organs. In most cases, the tumor and OARs delineated on MIP scans can have better coverage than 3D and AVG scans. However, when the repeatability of the patient’s respiratory cycle is poor and the breathing motion is irregular, the esophagus motion determined by MIP may have a large displacement. Previous research shows that the maximum radial error of MIP may exceed 1 cm (14). Our study also found that ESO_MIP_ is 24.69% smaller than U4D_ESO_, and the distances between them are −0.34, 2.32, −2.35, 0.10, −2.91, and 1.16 cm on the right, left, anterior, posterior, superior, and inferior aspects, respectively. U4D can cover the full extent of motion of the esophagus, which provides data support for the establishment of esophagus IRV and is conducive to the implementation of individualized precise radiotherapy for lung cancer patients.

Some scholars have studied the margins of heart PRV in patients with left breast cancer (15) and lung cancer (16). There are few studies on the margins of the esophagus PRV. Nardone et al. (9) studied the IRV of patients undergoing SBRT radiotherapy in the chest, which included the esophagus, but did not involve the study of the margin. Many studies are on the range of motion of the target area and the outer edge of the target area in esophagus cancer (17, 18).

Limitations of the study: since the heavy workload, only one experienced expert was invited to complete the outline of the esophagus. Daily imaging is required to calculate the random component to fully derive the PRV. In addition, the difference in breathing patterns between planned and actual treatments also requires 4D daily imaging to achieve. Therefore, the margin of this research report will increase after taking random errors into consideration.

## 5 Conclusion

In this study, we estimated the full extent of esophagus motion based on 4D CT scans of lung cancer patients. Moreover, we used this full extent of motion to derive margins accounting for systematic delineation errors for delineations based on 3D, AVG, and MIP scans. In general, MIP requires the smallest extension, while 3DCT requires the largest extension. After further validation and toxicity assessment, these margins could be the basis of a margin system for departments without access to full 4D CT scans.

## Data Availability Statement

The original contributions presented in the study are included in the article/supplementary material. Further inquiries can be directed to the corresponding author.

## Ethics Statement

The studies involving human participants were reviewed and approved by the Ethics Committee of Shanghai Chest Hospital (the committee’s reference Number: KS1974). The patients/participants [legal guardian/next of kin] provided written informed consent to participate in this study.

## Author Contributions

AF was involved in conceptualization, investigation, methodology, software, data curation, writing original draft preparation, and review/editing. HG was involved in data curation, writing review, and editing. HC was involved in conceptualization, methodology, and software. YS was involved in data curation and visualization. HW was involved in software, writing review, and editing. YD was involved in methodology and visualization. YH was involved in software. ZX and TZ were involved in conceptualization, methodology, project administration, supervision, writing original draft preparation, and review/editing. All authors contributed to the article and approved the submitted version.

## Funding

This work was supported by Shanghai Hospital Development Center “Clinical Research Plan of SHDC” (16CR3056A).

## Conflict of Interest

The authors declare that the research was conducted in the absence of any commercial or financial relationships that could be construed as a potential conflict of interest.

## Publisher’s Note

All claims expressed in this article are solely those of the authors and do not necessarily represent those of their affiliated organizations, or those of the publisher, the editors and the reviewers. Any product that may be evaluated in this article, or claim that may be made by its manufacturer, is not guaranteed or endorsed by the publisher.

## Appendix A

The code for collecting voxel coordination from dicom files.

i=dicominfo(‘AIP.dcm’);

i1=struct2cell(i.ROIContourSequence.Item_1.ContourSequence);

S=[];

for y=1:length(i1)

x1=[];y1=[];z1=[];g=[];q=[];A=[];B=[];

for j=1:3: length(i1{y,1}.ContourData)-2

x1=[x1; i1{y,1}.ContourData(j)];

end

for j=2: 3:length(i1{y,1}.ContourData)-1

y1=[y1; i1{y,1}.ContourData(j)];

end

for j=3: 3:length(i1{y,1}.ContourData)

z1=[z1; i1{y,1}.ContourData(j)];

end

d=[x1 y1];

d1=sortrows(d,2);

[m,n]=size(d1);

for i=1:m-1

if d1(i,2)==d1(i+1,2)

f=round(abs(d1(i,1)- d1(i+1,1))/0.9765625);

for j=1:f

q(j,1)=min(d1(i:i+1,1)) +(j-1)*0.9765625;

q(j,2)=d1(i,2);

end

else

end

A=[A;q];

end

A1=unique(A,’row’);

A1=sortrows(A1,2);

for j=1:length(A1)-1

if A1(j,2)==A1(j+1,2) && abs(A1(j,1)-A1(j+1,1))<0.9765625

B=[B;A1(j),:];

else

end

end

D=setxor(A1,B,’rows’);

for j=1:length(D)

g(j,1)= z1(3,1);

end

S=[S; D g];

End

## Appendix B

**Table E1:** Margin proposed by McKenzie [Ref (12)].

Margin 1D	Margin 2D	Margin 3D
**Equation**		
1.3Σ+0.5σ+M	2.2Σ+0.5σ+M	2.5Σ+0.5σ+M
**Situation**		
Simple dose distributions where the ‘threat’ of high dose region comes from one direction.	For dose distributions where the ‘threat’ of high dose region comes from two directions.	Most complex situations where the ‘threat’ of high dose region is comes from three directions.
